# Surgical Versus Medical Management of Necrotizing Enterocolitis With and Without Intestinal Perforation: A Retrospective Chart Review

**DOI:** 10.7759/cureus.15722

**Published:** 2021-06-17

**Authors:** Muhammad Khalid Syed, Ahmad A Al Faqeeh, Noman Saeed, Talal Almas, Tarek Khedro, Muhammad Ali Niaz, M. Ali Kanawati, Salman Hussain, Hussain Mohammad, Lamees Alshaikh, Lina Alshaikh, Abdulaziz Abdulhadi, Abdulaziz Alshamlan, Saifullah Syed, Hamdy Katar Hanafi Mohamed

**Affiliations:** 1 Pediatric Surgery, King Fahad Hospital, Al Baha, SAU; 2 Neonatology, King Fahad Hospital, Al Baha, SAU; 3 Internal Medicine, Royal College of Surgeons in Ireland, Dublin, IRL; 4 Neonatology, Kind Fahad Hospital, Al Baha, SAU

**Keywords:** necrotizing enterocolitis, pediatric ileostomy, intestinal perforation, metronidazole, abdominal sepsis

## Abstract

Background

Necrotizing enterocolitis (NEC) is a debilitating disease that predominantly afflicts premature neonates, although it can also affect term neonates. The clinical features of the ailment vary widely and range from transient feed intolerance to life-threatening complications such as septicemia and disseminated intravascular coagulation. While surgery is usually only reserved for severe cases, such as those presenting with intestinal perforation, the role of surgical management in cases of NEC without perforation remains elusive.

Methods

A retrospective chart review of patients, three years in duration, was conducted and studied confirmed cases of NEC. The clinical presentations studied included cases of NEC with pneumatosis intestinalis, fixed bowel loop, pneumoperitoneum, and abdominal wall erythema. The patients were divided with regards to their intestinal perforation status and with pertinence to the treatment modality employed (medical or surgical). The results in either group were eventually analyzed in terms of the overall survival rate.

Results

A total of 48 patients were included in the study, of which 79.16% presented without perforation and 20.83% with perforation. Of the study participants included, 26 were females and 22 were males. Pertinently, no gender predominance was appreciated. In patients without perforation, medical management was noted to boast a lower mortality rate when compared with surgical intervention (15.6% vs 50.0%, respectively). In patients with perforation, the overall mortality was noted to hover at 50.0%, which was higher than that encountered in the non-perforation group.

Conclusion

In patients with NEC without perforation, surgical treatment confers no comparative therapeutic advantage when compared with medical management alone. Conservative management with broad-spectrum antibiotics including metronidazole yields equally favorable outcomes in such cases.

## Introduction

Necrotizing enterocolitis (NEC), which primarily affects premature neonates, is the most common gastrointestinal emergency in neonates [1]. NEC boasts a high morbidity and mortality rate and thus remains at the forefront of a controversy with regards to the absolute indications of surgical management of NEC. The controversy lies in identifying the pre-operative factors, such as patient comorbidities, to determine whether a surgical intervention is more superior to medical management, and the specific type of surgical intervention that confers the most optimal therapeutic advantage [1,2]. The preferred method of surgical intervention for NEC with perforation remains controversial, with the two most frequently used procedures being laparotomy and primary peritoneal drainage [2]. Literature has suggested that the most optimal time for intervention through either medical or surgical management is after the onset of severe ischemia but before perforation has occurred [2]. However, in clinical practice, identifying this timeframe for optimal intervention is extremely challenging [2]. To date, several indications for performing a surgical intervention for NEC, including pneumoperitoneum, remarkable paracentesis, and the presence of portal venous gas (PVG), have been identified [3]. Notably, evidence of pneumoperitoneum, indicating the presence of intestinal perforation, is considered the only absolute indication for surgical intervention [2]. Contrarily, the relative indications for surgical management include factors such as clinical deterioration and pneumatosis [2]. 

While the presence of PVG is not traditionally considered an absolute indication for surgery, several studies have now suggested that PVG, as observed on abdominal radiographs, is considered a poor prognosticator, and therefore warrants early surgical intervention [4,5]. Cikrit et al. reported a 71% mortality rate in cases where PVG was evident on abdominal radiographs [4,5]. In contrast, other studies have reported that patients with PVG may recover without surgical intervention, consistent with findings in our study [6,7]. Moss and colleagues demonstrated that there was no difference in survival between patients who underwent either surgical procedure while other studies have divulged a more favorable outcome with laparotomy in comparison to primary peritoneal drainage [8-10]. The aim of the present study is to ascertain whether medical or surgical management confers a comparative therapeutic advantage in cases of NEC with and without perforation.

## Materials and methods

The present retrospective cross-sectional study was conducted in the neonatal intensive care unit (NICU) over a course of three years. Patient charts were perused for a confirmed diagnosis of NEC with and without intestinal perforation. The inclusion criteria adopted ensured that all cases encompassing pneumatosis intestinalis, presence of PVG, fixed bowel loop, and abdominal wall erythema were included. The exclusion criteria adopted in the present study meant that suspected cases of NEC without the aforementioned features were excluded from the eventual analysis. Furthermore, the parameters studied included the clinical signs, the symptomatology, and the associated congenital malformations. Additional data pertaining to the feed type, maternal illness, and the medications used were also collated. Investigative workup results, such as the platelet count, coagulation profile, blood culture, and blood gas analysis, were also included and studied. These data were correlated with the ultrasound and abdominal radiograph findings. Once the data were collated, the proportion of patients managed with and without surgical intervention was ascertained. In cases requiring surgical management, the type and nature of the intervention was documented. The patient outcomes were assessed in terms of their survival status. The collated data was eventually analyzed using Statistical Package for Social Sciences (SPSS), version 23.0 software (IBM Corporation, Armonk, NY).

## Results

The current study analyzed the charts of 48 patients with a clinically confirmed diagnosis of NEC who presented to the hospital within a three-year period. All of the patients included were neonates, with most presenting with symptoms in the second week of life. Of the 48 patients studied, 38 (79.16%) manifested with features of NEC without perforation, while 10 (20.83%) patients presented with intestinal perforation. The study subjects were studied with pertinence to their intestinal perforation status, and appropriate management was thus instituted in accordance with their status.

Out of 38 patients who had no perforation but did demonstrate other mentioned features of NEC, ileostomy was performed in six patients out of which three patients, constituting 50% of this subgroup, died while the other half survived. The remaining 32 patients were managed medically with intravenous metronidazole therapy. Amongst these patients who were managed conservatively, 15.6% died while 84.4% survived, purporting a higher survival rate than in the surgical management subgroup. These outcomes are tabulated in Table 1. 

**Table 1:** The treatment modality employed and the outcomes in patients with NEC without intestinal perforation. NEC: necrotizing enterocolitis.

NEC without perforation	Total (N)	Treatment	Total (N)	Survival status	Total (N)
	38	Ileostomy	6	Survived	3
				Died	3
		Medically treated	32	Survived	27
				Died	5

Within the present study, 10 patients presented with intestinal perforation. Of these patients, peritoneal drain was placed in six unstable patients while surgical intervention, through the means of an ileostomy, was performed in four stable patients. The outcomes within this perforation subgroup are elucidated in Table 2. 

**Table 2:** The treatment modality employed and the outcomes in patients with NEC with intestinal perforation. NEC: necrotizing enterocolitis.

NEC with perforation	Total (N)	Treatment	Total (N)	Survival status	Total (N)
	10	Peritoneal drain	3	Survived	1
				Died	2
		Peritoneal drain followed by ileostomy	3	Survived	1
				Died	2
		Laparotomy/ileostomy	4	Survived	3
				Died	1

As can be gauged from Table 2, six patients presenting with NEC with perforation underwent peritoneal drain placement. Of these six patients, one patient demonstrated a perforation that sealed spontaneously, thereby discounting the need for further operation. Two patients, accounting for 33.3% of patients in this subgroup, died due to sepsis. The remaining three patients demonstrated clinical amelioration to some extent and were therefore able to undergo laparotomy. However, post ileostomy, two of these patients survived while one patient ultimately succumbed to the complications and died. Furthermore, of the four patients who underwent laparotomy/ileostomy, 75% survived while the mortality rate hovered at 25% within this subgroup.

**Figure 1:**
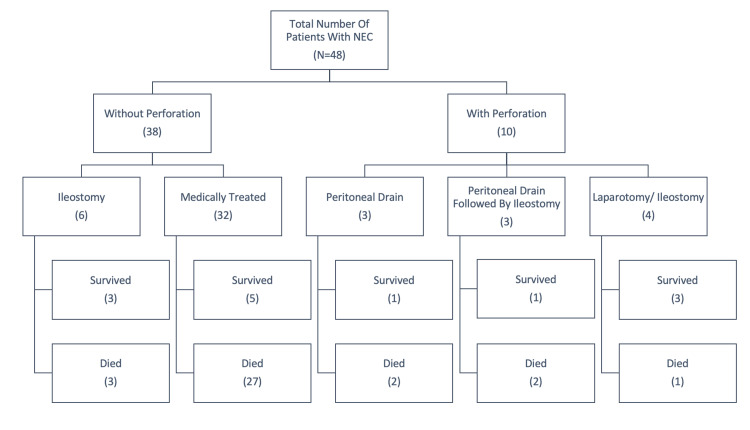
A flowchart depicting the perforation status, the treatment modality employed, and the eventual outcomes within our study cohort. NEC: necrotizing enterocolitis.

A detailed breakdown of the study subjects in accordance with their perforation status, the treatment modality employed, and the eventual outcomes are delineated in Figure 1. 

## Discussion

NEC is an inflammatory disease of the bowel [11]. It is predominantly a disease of premature infants, with 90% of cases noted to occur in preterm neonates [12]. However, full-term neonates are still at risk, accounting for the remaining 10% of the cases [12]. Other causes of NEC include a low birth weight, formula feeding, sepsis, extended use of antibiotics, indomethacin therapy to promote closure of a patent ductus arteriosus (PDA), and glucocorticoid therapy [13-15]. Maternal factors include chorioamnionitis, antenatal cocaine use, antenatal maternal infection, preeclampsia, and smoking, although these risk factors have not been as consistently implicated in NEC development as the others [13,16-18]. Breastfeeding has been found to be the most effective protective measure due to its provision of immunological factors that promote microbiota formation in the neonate’s gastrointestinal tract [19]. However, this parameter was not evaluated in the present study. 

The pathophysiology underlying NEC involves a disruption of the normal gut flora, a process known as dysbiosis, and colonization of pathogenic bacteria that lead to invasion and disruption of the gut mucosa [20,21]. Pro-inflammatory chemical mediators, such as cytokines and chemokines, and the presence of bacterial toxins together elicit a potent inflammatory process that ultimately results in intestinal necrosis that, if left untreated, progresses to life-threatening complications such as septicemia and disseminated intravascular coagulation (DIC) [19,22]. Interestingly, infants with surgically indicated NEC were found to have a loss of Paneth cells, which reside in the crypts of Lieberkühn in the small and large intestines and play a major role in the maintenance of a healthy microbiota as well as antimicrobial defense [23]. 

**Figure 2:**
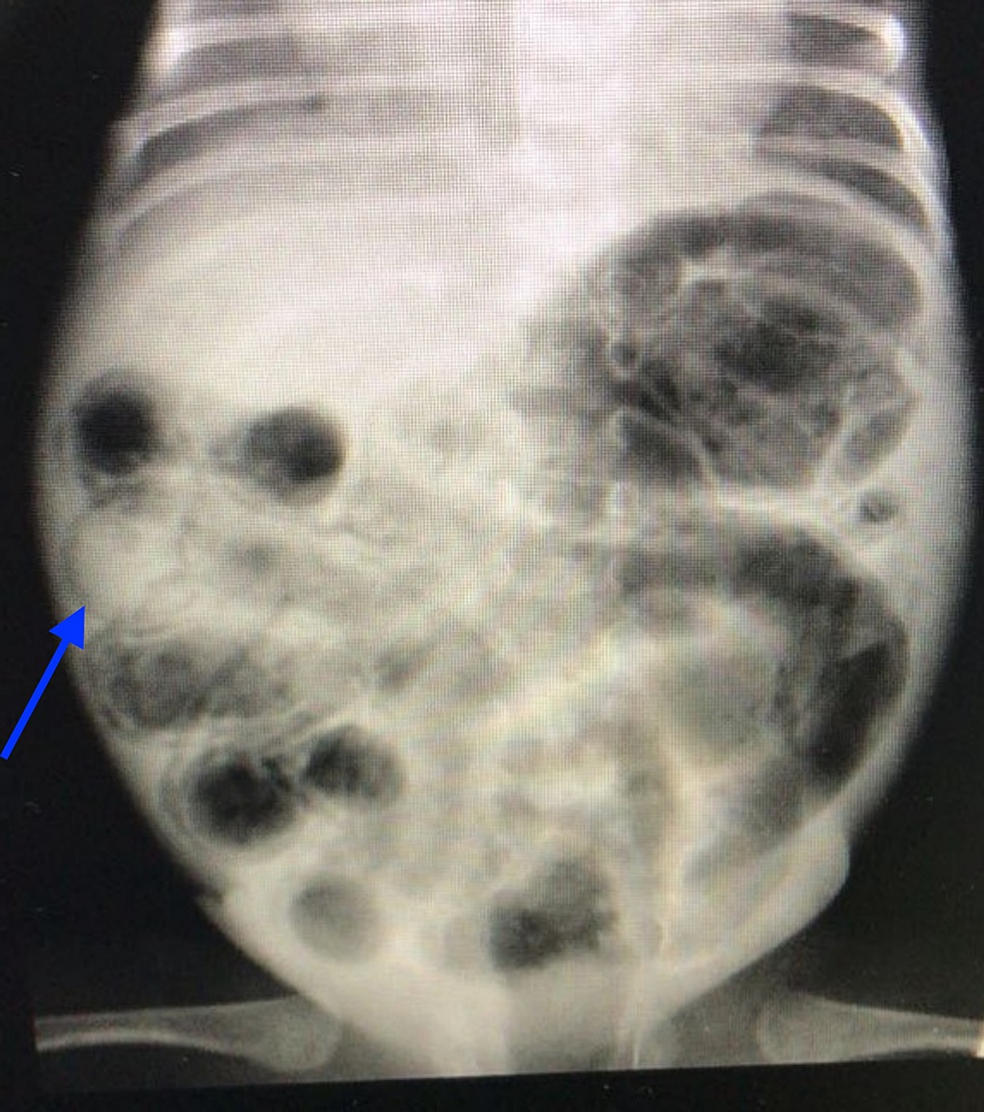
A radiological depiction of pneumatosis intestinalis (blue arrow) as observed in the study population.

**Figure 3:**
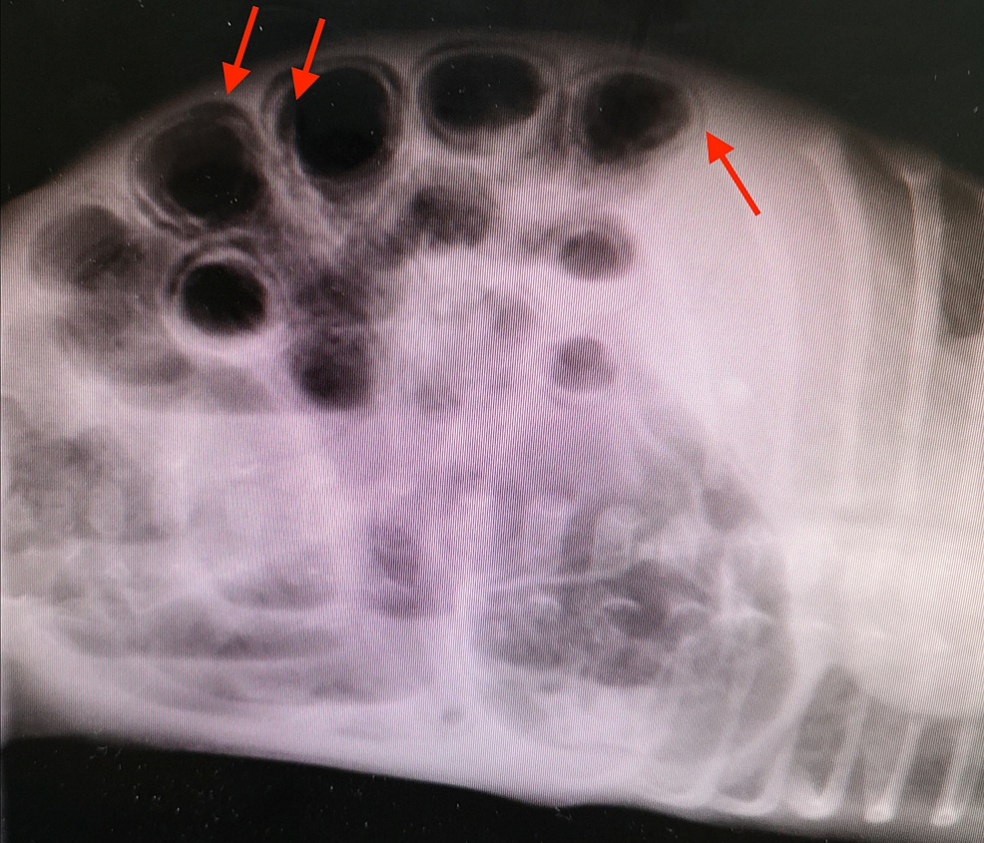
A radiological depiction of pneumatosis intestinalis (red arrows) as observed in the study population.

The cost of NEC management is exorbitantly high, accounting for approximately 20% of the total costs incurred by the NICUs annually [12]. The total annual estimated cost in the United States is between $500 million to $1 billion [24]. In severe cases, the necessitation and urgency of surgery only increase this expense, especially considering that survivors often remain in the NICU for more than 90 days and not uncommonly for up to 6 months [12]. The modified Bell’s Staging Criteria is used for the management of the disease [15]. In the early stages of NEC, common signs in the neonate include an aversion to feeding, lethargy, and mild abdominal distension [15]. In these cases, supportive treatment is indicated, often involving a cessation of feeding, nasogastric tube aspiration, and intravenous antibiotics [24]. Disease progression leads to the development of characteristic radiological features such as pneumatosis intestinalis, PVG, fixed bowel loop, and apparent abdominal mass or distension and abdominal wall erythema. The radiological findings consistent with pneumatosis intestinalis, as observed in our study population, are delineated in Figure 2 and Figure 3. 

**Figure 4:**
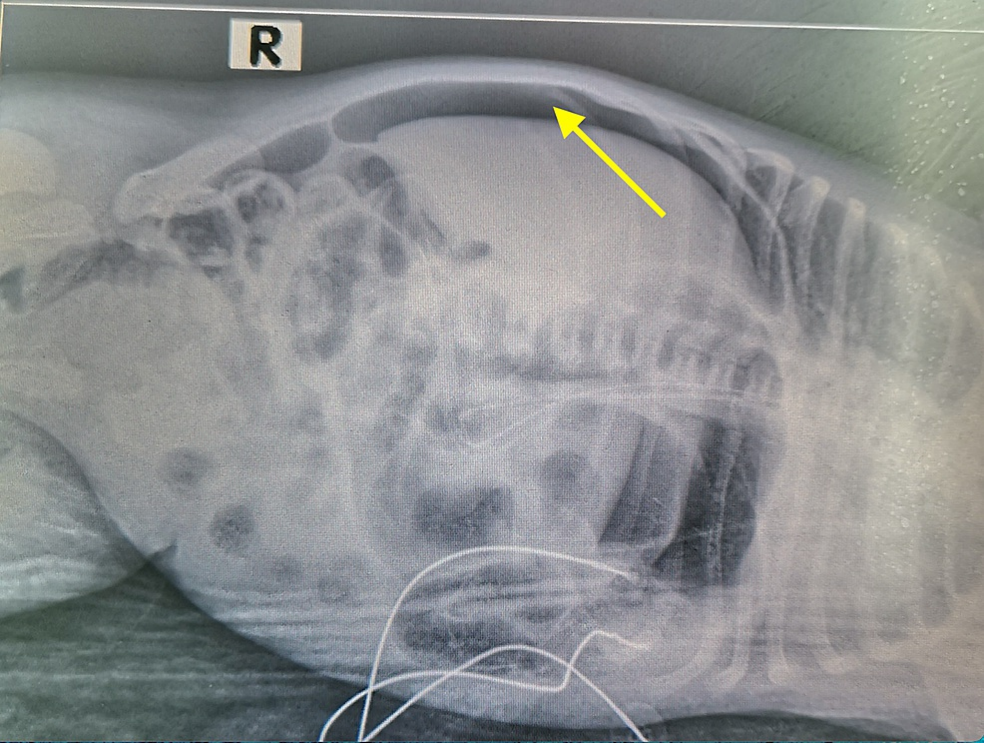
A lateral cross-table radiograph showing free gas above the liver (yellow arrow), which is suggestive of intestinal perforation.

Blood investigations will typically indicate thrombocytopenia, leukopenia, electrolyte imbalance with a metabolic acidosis, and a prolonged coagulation profile. Supportive treatment in this setting is amplified to include total parenteral nutrition, oxygenation and mechanical ventilation, and broad-spectrum antibiotics, in conjunction with metronidazole, to ensure coverage of both anaerobic and aerobic bacteria [24]. Early surgical intervention at this point is often recommended since further progression results in intestinal perforation, mandating the need for surgical intervention. Intestinal perforation encountered in one of the patients is elucidated in Figure 4.

Depending on the stability of the patient, surgical intervention involves either peritoneal drain placement or a direct laparotomy [1,24-25]. Unstable patients usually undergo peritoneal drain insertion, while some perforations can heal spontaneously [26]. However, most will require a laparotomy once they have stabilized. Among laparotomy procedures, unifocal perforation necessitates resection and primary anastomosis, whereas multifocal perforation, pan-intestinal involvement, or patients who have yet to stabilize typically require an ileostomy [27].

The purpose of the current three-year retrospective study was to present the results of surgical and non-surgical management of 48 neonates with NEC and compare it against the literature. In our study, where early surgical intervention was performed in the absence of perforation, but the presence of other relative indications, no benefit in overall survival was observed. Medical management with the addition of metronidazole was sufficient in the treatment of patients with NEC without signs of perforation. Our data suggest that the gold standard procedure for NEC with perforation is laparotomy with surgical resection and ileostomy, and primary peritoneal drainage should only be performed if the patient is too unstable to undergo a laparotomy. Furthermore, patients who underwent primary peritoneal drainage will likely require laparotomy once stabilized, as can be gauged from the present study.

## Conclusions

In patients presenting with NEC without perforation, surgical management may confer no additional therapeutic advantage. In such cases, conservative management through the means of broad-spectrum antibiotics, with the addition of metronidazole, portends equally favorable outcomes while circumventing the complications that are routinely associated with surgical interventions.

## References

[1] Pierro A (2005). The surgical management of necrotising enterocolitis. Early Hum Dev.

[2] Henry MC, Lawrence Moss R (2005). Surgical therapy for necrotizing enterocolitis: bringing evidence to the bedside. Semin Pediatr Surg.

[3] Kosloske AM (1994). Indications for operation in necrotizing enterocolitis revisited. J Pediatr Surg.

[4] Cikrit D, Mastandrea J, Grosfeld JL, West KW, Schreiner RL (1985). Significance of portal vein air in necrotizing entercolitis: analysis of 53 cases. J Pediatr Surg.

[5] Ein SH, Marshall DG, Girvan D (1977). Peritoneal drainage under local anesthesia for perforations from necrotizing enterocolitis. J Pediatr Surg.

[6] Tam AL, Camberos A, Applebaum H (2002). Surgical decision making in necrotizing enterocolitis and focal intestinal perforation: predictive value of radiologic findings. J Pediatr Surg.

[7] Sharma R, Tepas JJ 3rd, Hudak ML (2005). Portal venous gas and surgical outcome of neonatal necrotizing enterocolitis. J Pediatr Surg.

[8] Moss RL, Dimmitt RA, Barnhart DC (2006). Laparotomy versus peritoneal drainage for necrotizing enterocolitis and perforation. N Engl J Med.

[9] Horwitz JR, Lally KP, Cheu HW, Vazquez WD, Grosfeld JL, Ziegler MM (1995). Complications after surgical intervention for necrotizing enterocolitis: a multicenter review. J Pediatr Surg.

[10] Rees CM, Eaton S, Kiely EM, Wade AM, McHugh K, Pierro A (2008). Peritoneal drainage or laparotomy for neonatal bowel perforation? A randomized controlled trial. Ann Surg.

[11] Shulhan J, Dicken B, Hartling L, Larsen BMK (2017). Current knowledge of necrotizing enterocolitis in preterm infants and the impact of different types of enteral nutrition products. Adv Nutr.

[12] Gephart SM, McGrath JM, Effken JA, Halpern MD (2012). Necrotizing enterocolitis risk: state of the science. Adv Neonatal Care.

[13] Alganabi M, Lee C, Bindi E, Li B, Pierro A (2019). Recent advances in understanding necrotizing enterocolitis. F1000Res.

[14] Travers CP, Clark RH, Spitzer AR, Das A, Garite TJ, Carlo WA (2017). Exposure to any antenatal corticosteroids and outcomes in preterm infants by gestational age: prospective cohort study. BMJ.

[15] Gregory KE, Deforge CE, Natale KM, Phillips M, Van Marter LJV (2011). Necrotizing enterocolitis in the premature infant: neonatal nursing assessment, disease pathogenesis, and clinical presentation. Adv Neonatal Care.

[16] Been JV, Lievense S, Zimmermann LJ, Kramer BW, Wolfs TG (2013). Chorioamnionitis as a risk factor for necrotizing enterocolitis: a systematic review and meta-analysis. J Pediatr.

[17] Czyrko C, Del Pin CA, O'Neill JA Jr (1991). Maternal cocaine abuse and necrotizing enterocolitis: outcome and survival. J Pediatr Surg.

[18] Downard CD, Grant SN, Maki AC (2012). Maternal cigarette smoking and the development of necrotizing enterocolitis. Pediatrics.

[19] Schnabl KL, Van Aerde JE, Thomson AB, Clandinin MT (2008). Necrotizing enterocolitis: a multifactorial disease with no cure. World J Gastroenterol.

[20] Elgin TG, Kern SL, McElroy SJ (2016). Development of the neonatal intestinal microbiome and its association with necrotizing enterocolitis. Clin Ther.

[21] Denning NL, Prince JM (2018). Neonatal intestinal dysbiosis in necrotizing enterocolitis. Mol Med.

[22] De Plaen IG (2013). Inflammatory signaling in necrotizing enterocolitis. Clin Perinatol.

[23] Zhang C, Sherman MP, Prince LS, Bader D, Weitkamp JH, Slaughter JC, McElroy SJ (2012). Paneth cell ablation in the presence of Klebsiella pneumoniae induces necrotizing enterocolitis (NEC)-like injury in the small intestine of immature mice. Dis Model Mech.

[24] Neu J, Walker WA (2011). Necrotizing enterocolitis. N Engl J Med.

[25] Zangari A, Gulia C, Piergentili R (2017). Indications to surgery in necrotising enterocolitis of extremely low birth weight prematures. Can we do better?. J Pediatr Neonatal Care.

[26] Thakkar HS, Lakhoo K (2016). The surgical management of necrotising enterocolitis (NEC). Early Hum Dev.

[27] Zani A, Eaton S, Puri P (2015). International survey on the management of necrotizing enterocolitis. Eur J Pediatr Surg.

